# Chlorido[(*E*)-2-hydr­oxy-6-(isonicotinoyl­hydrazonometh­yl)phen­yl]mercury(II) monohydrate

**DOI:** 10.1107/S1600536809023824

**Published:** 2009-06-27

**Authors:** Su-Zhen Bai, Xin-Hua Lou, Hong-Mei Li, Hui Shi

**Affiliations:** a College of Chemistry and Chemical Engineering, Pingdingshan University, Pingdingshan 467002, People’s Republic of China; b College of Chemistry and Chemical Engineering, Luoyang Normal University, Luoyang 471022, People’s Republic of China; c Chemical Engineering and Pharmaceutics School, Henan University of Science and Technology, Luoyang 471003, People’s Republic of China

## Abstract

The asymmetric unit of the title compound, [Hg(C_13_H_10_N_3_O_2_)Cl]·H_2_O, contains two independent mercury(II) complexes with slightly different conformations, related *via* a pseudo-inversion centre, and two water mol­ecules. The Hg^II^ atoms show a typical linear geometry to a C atom of the benzene ring and to a Cl atom. A benzene C and the azomethine N atom chelate the Hg^II^ atoms with weak intra­molecular Hg⋯N bonding distances of 2.735 (3) and 2.739 (3) Å, respectively. The resulting five-membered metallacycles are nearly coplanar with the benzene rings [dihedral angles = 0.9 (1) and 0.7 (1)°], while the pyridine rings make dihedral angles with the benzene units of 58.17 (1) and 56.58 (1)°. In the crystal structure, the Hg^II^ complexes are linked by hydr­oxy donor and pyridine acceptor groups into chains along [010]. The water mol­ecules connect the complexes through inter­molecular O—H⋯O_carbon­yl_ bonds in the *a*-axis direction, and the azomethine H atoms donate towards the water O atoms, forming a three-dimensional network of inter­molecular O—H⋯N, O—H⋯O and N—H⋯O hydrogen bonds.

## Related literature

For general background, see: Gruter *et al.* (1995 ▶); Soro *et al.* (2005 ▶); Xu *et al.* (2009*b* ▶). For related structures, see: Hao *et al.* (2007 ▶); Lin *et al.* (2002 ▶); For the synthesis of related cyclomercurated compounds, see: Xu *et al.* (2009*a* ▶).
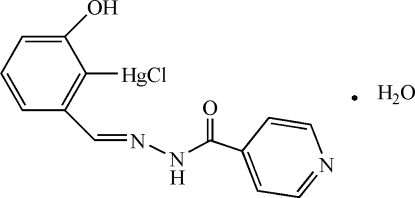


## Experimental

### 

#### Crystal data

[Hg(C_13_H_10_N_3_O_2_)Cl]·H_2_O
*M* *_r_* = 494.30
Monoclinic, 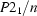

*a* = 14.5932 (16) Å
*b* = 14.0111 (15) Å
*c* = 15.3711 (17) Å
β = 104.6850 (10)°
*V* = 3040.2 (6) Å^3^
*Z* = 8
Mo *K*α radiation
μ = 10.31 mm^−1^
*T* = 296 K
0.37 × 0.28 × 0.25 mm

#### Data collection

Bruker SMART APEX CCD area-detector diffractometer
Absorption correction: multi-scan (*SADABS*; Sheldrick, 1996 ▶) *T*_min_ = 0.113, *T*_max_ = 0.179 (expected range = 0.048–0.076)
22798 measured reflections
5658 independent reflections
4683 reflections with *I* > 2σ(*I*)
*R*_int_ = 0.030

#### Refinement

*R*[*F* ^2^ > 2σ(*F* ^2^)] = 0.021
*wR*(*F* ^2^) = 0.046
*S* = 1.05
5658 reflections
381 parameters
H-atom parameters constrained
Δρ_max_ = 0.52 e Å^−3^
Δρ_min_ = −0.83 e Å^−3^

### 

Data collection: *SMART* (Bruker, 2004 ▶); cell refinement: *SAINT* (Bruker, 2004 ▶); data reduction: *SAINT*; program(s) used to solve structure: *SHELXS97* (Sheldrick, 2008 ▶); program(s) used to refine structure: *SHELXL97* (Sheldrick, 2008 ▶); molecular graphics: *SHELXTL* (Sheldrick, 2008 ▶); software used to prepare material for publication: *SHELXTL* and *PLATON* (Spek, 2009 ▶).

## Supplementary Material

Crystal structure: contains datablocks global, I. DOI: 10.1107/S1600536809023824/si2184sup1.cif

Structure factors: contains datablocks I. DOI: 10.1107/S1600536809023824/si2184Isup2.hkl

Additional supplementary materials:  crystallographic information; 3D view; checkCIF report

## Figures and Tables

**Table 1:** Hydrogen-bond geometry (Å, °)

*D*—H⋯*A*	*D*—H	H⋯*A*	*D*⋯*A*	*D*—H⋯*A*
O6—H4*W*⋯O2^i^	0.83	2.15	2.898 (4)	150
O6—H3*W*⋯O3	0.83	2.30	3.023 (4)	146
O5—H2*W*⋯O1	0.83	2.17	2.963 (4)	159
O5—H1*W*⋯O4^ii^	0.84	2.06	2.876 (4)	166
N5—H5*D*⋯O6^iii^	0.86	2.04	2.872 (4)	162
N2—H2*D*⋯O5^iv^	0.86	2.06	2.890 (4)	161
O3—H3⋯N6^v^	0.82	1.92	2.737 (4)	171
O1—H1⋯N3^vi^	0.82	1.93	2.733 (4)	167

Symmetry codes: (i) 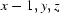; (ii) 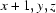; (iii) 
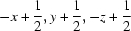
; (iv) 
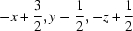
; (v) 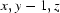; (vi) 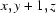.

**Fig. 1.**
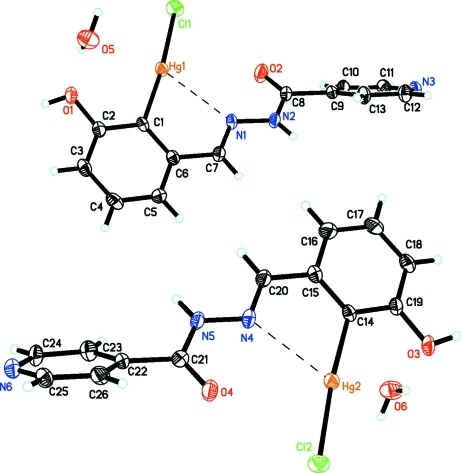
The molecular structure of the title compound with displacement ellipsoids at the 30% probability level.

## supplementary crystallographic information

### Comment

Cyclometallated compounds have attracted much research interest owing to theirs utility in synthesis, catalysis and materials (Gruter *et al.*, 1995; Xu *et al.*, 2009*b*). Among them, cyclomercurated compounds are easy to prepare through a C—H activation process and are stable but reasonably reactive. Although numerous cyclomercurated compounds have been widely investigated, and many examples have been reported(Soro *et al.*, 2005; Hao *et al.*, 2007), only a few cyclometallated Schiff bases containing heterocyclic ring are known(Lin *et al.*, 2002).

The asymmetric unit of the title compound (Fig.1) contains two independent mercury(II) complexes with slightly different conformations, related *via* a pseudo-inversion centre (1/2*a*, 3/4*b*, 1/4*c*), and two water molecules. The Hg^II^ atoms show a typical linear coordination geometry with a carbon atom of the benzene ring and the chloride atom in *trans* position. A benzene carbon and the azomethine nitrogen atom chelate the mercury(II) atoms with weak intramolecular Hg···N bonding distances of 2.735 (3)Å and 2.739 (3) Å. which are shorter than those of the related Hg^II^ complex (Hao *et al.*, (2007); Lin *et al.*, (2002); Xu *et al.*, (2009a). The C—Hg and Hg—Cl bond distances are within normal ranges. The C1—Hg1—Cl1 and C14—Hg2—Cl2 angles are 173.85 (10)° and 174.67 (10)°, slightly smaller than the ideal value of 180° in organic derivatives of mercury. The resulting five-membered metallacycles are nearly coplanar with the benzene ring, while the pyridine are not coplanar with the benzene. In the crystal structure, intermolecular O—H···O, N—H···O and O—H···N hydrogen bonds (Table 1) link the independent Hg^II^ complexes and the water molecules into a three-dimensional network.

### Experimental

Chlorido(2-formyl-6-hydroxybenzaldehyde-*kC^1^*)mercury(II) was synthesized according to the reported procedure (Xu *et al.*, 2009*a*). The title compound was prepared from the above compound with isonicotinoylhydrazine and recrystallized from ethanol solution at room temperature to give the desired product as colourless crystals suitable for single-crystal X-ray diffraction.

### Refinement

All H atoms were placed in geometrically idealized positions and constrained to ride on their patent atoms, with distances: C—H = 0.93 Å, N—H = 0.86 Å, and O—H = 0.82 Å. The *U*_iso_(H) values were set at 1.2*U*_eq_ (C,*N*) and 1.5*U*_eq_(O).

### Crystal data

**Table:**

[Hg(C_13_H_10_N_3_O_2_)Cl]·H_2_O	*F*(000) = 1856
*M**_r_* = 494.30	*D*_x_ = 2.160 Mg m^−^^3^
Monoclinic, *P*2_1_/*n*	Mo *K*α radiation, λ = 0.71073 Å
*a* = 14.5932 (16) Å	Cell parameters from 5064 reflections
*b* = 14.0111 (15) Å	θ = 2.7–28.3°
*c* = 15.3711 (17) Å	µ = 10.31 mm^−^^1^
β = 104.685 (1)°	*T* = 296 K
*V* = 3040.2 (6) Å^3^	Block, colorless
*Z* = 8	0.37 × 0.28 × 0.25 mm

### Data collection

**Table:**

Bruker SMART APEX CCD area-detector diffractometer	5658 independent reflections
Radiation source: fine-focus sealed tube	4683 reflections with *I* > 2σ(*I*)
graphite	*R*_int_ = 0.030
φ and ω scans	θ_max_ = 25.5°, θ_min_ = 2.2°
Absorption correction: multi-scan (*SADABS*; Sheldrick, 1996)	*h* = −17→17
*T*_min_ = 0.113, *T*_max_ = 0.179	*k* = −16→16
22798 measured reflections	*l* = −16→18

### Refinement

**Table:**

Refinement on *F*^2^	Primary atom site location: structure-invariant direct methods
Least-squares matrix: full	Secondary atom site location: difference Fourier map
*R*[*F*^2^ > 2σ(*F*^2^)] = 0.021	Hydrogen site location: inferred from neighbouring sites
*wR*(*F*^2^) = 0.046	H-atom parameters constrained
*S* = 1.05	*w* = 1/[σ^2^(*F*_o_^2^) + (0.0197*P*)^2^ + 1.3364*P*] where *P* = (*F*_o_^2^ + 2*F*_c_^2^)/3
5658 reflections	(Δ/σ)_max_ = 0.002
381 parameters	Δρ_max_ = 0.52 e Å^−^^3^
0 restraints	Δρ_min_ = −0.83 e Å^−^^3^

### Special details

**Table:**

Geometry. All e.s.d.'s (except the e.s.d. in the dihedral angle between two l.s. planes)are estimated using the full covariance matrix. The cell e.s.d.'s are takeninto account individually in the estimation of e.s.d.'s in distances, anglesand torsion angles; correlations between e.s.d.'s in cell parameters are onlyused when they are defined by crystal symmetry. An approximate (isotropic)treatment of cell e.s.d.'s is used for estimating e.s.d.'s involving l.s. planes.
Refinement. Refinement of *F*^2^ against ALL reflections. The weighted *R*-factor *wR* and goodness of fit *S* are based on *F*^2^, conventional *R*-factors *R* are based on *F*, with *F* set to zero for negative *F*^2^. The threshold expression of *F*^2^ > σ(*F*^2^) is used only for calculating *R*-factors(gt) *etc*. and is not relevant to the choice of reflections for refinement. *R*-factors based on *F*^2^ are statistically about twice as large as those based on *F*, and *R*- factors based on ALL data will be even larger.

### Fractional atomic coordinates and isotropic or equivalent isotropic displacement parameters (Å^2^)

**Table:**

	*x*	*y*	*z*	*U*_iso_*/*U*_eq_	
Hg1	0.909422 (9)	0.795528 (10)	0.140566 (10)	0.03271 (5)	
Hg2	0.088755 (10)	0.667392 (10)	0.363229 (10)	0.03354 (5)	
Cl1	1.06292 (6)	0.74039 (8)	0.15848 (7)	0.0482 (3)	
Cl2	−0.06489 (7)	0.72341 (8)	0.34424 (7)	0.0485 (3)	
O1	0.85291 (17)	1.00819 (18)	0.13735 (19)	0.0444 (7)	
H1	0.8409	1.0641	0.1231	0.067*	
O2	0.91602 (18)	0.54581 (19)	0.0795 (2)	0.0539 (8)	
O3	0.14451 (18)	0.45504 (17)	0.3573 (2)	0.0473 (7)	
H3	0.1565	0.3987	0.3693	0.071*	
O4	0.08053 (18)	0.91980 (18)	0.4187 (2)	0.0494 (7)	
N1	0.7764 (2)	0.6537 (2)	0.1123 (2)	0.0339 (7)	
N2	0.7733 (2)	0.55515 (19)	0.11402 (19)	0.0338 (7)	
H2D	0.7253	0.5255	0.1239	0.041*	
N3	0.8374 (2)	0.2013 (2)	0.1131 (2)	0.0397 (8)	
N4	0.2225 (2)	0.8089 (2)	0.3911 (2)	0.0362 (7)	
N5	0.2266 (2)	0.9077 (2)	0.39086 (19)	0.0352 (7)	
H5D	0.2760	0.9368	0.3836	0.042*	
N6	0.1645 (2)	1.2624 (2)	0.3851 (2)	0.0397 (8)	
C1	0.7779 (2)	0.8579 (2)	0.1197 (2)	0.0279 (8)	
C2	0.7707 (3)	0.9568 (3)	0.1195 (2)	0.0349 (8)	
C3	0.6823 (3)	1.0005 (3)	0.1066 (3)	0.0431 (10)	
H3A	0.6779	1.0667	0.1076	0.052*	
C4	0.6011 (3)	0.9454 (3)	0.0924 (3)	0.0495 (11)	
H4	0.5422	0.9746	0.0830	0.059*	
C5	0.6075 (3)	0.8465 (3)	0.0920 (3)	0.0411 (9)	
H5	0.5528	0.8097	0.0830	0.049*	
C6	0.6953 (2)	0.8023 (2)	0.1051 (2)	0.0313 (8)	
C7	0.6986 (3)	0.6972 (2)	0.1046 (2)	0.0331 (8)	
H7	0.6430	0.6625	0.0986	0.040*	
C8	0.8484 (3)	0.5069 (2)	0.0996 (2)	0.0351 (9)	
C9	0.8433 (2)	0.4003 (2)	0.1063 (2)	0.0302 (8)	
C10	0.8804 (2)	0.3447 (2)	0.0486 (3)	0.0361 (9)	
H10	0.9089	0.3731	0.0075	0.043*	
C11	0.8744 (2)	0.2467 (3)	0.0532 (3)	0.0379 (9)	
H11	0.8973	0.2102	0.0127	0.046*	
C12	0.8025 (3)	0.2558 (3)	0.1688 (3)	0.0416 (9)	
H12	0.7760	0.2254	0.2104	0.050*	
C13	0.8035 (3)	0.3542 (3)	0.1680 (3)	0.0393 (9)	
H13	0.7782	0.3890	0.2079	0.047*	
C14	0.2197 (2)	0.6052 (2)	0.3792 (2)	0.0291 (8)	
C15	0.3026 (3)	0.6602 (2)	0.3951 (2)	0.0324 (8)	
C16	0.3903 (3)	0.6153 (3)	0.4047 (3)	0.0446 (10)	
H16	0.4453	0.6518	0.4149	0.054*	
C17	0.3955 (3)	0.5171 (3)	0.3992 (3)	0.0510 (11)	
H17	0.4540	0.4876	0.4057	0.061*	
C18	0.3143 (3)	0.4625 (3)	0.3840 (3)	0.0447 (10)	
H18	0.3180	0.3965	0.3798	0.054*	
C19	0.2271 (3)	0.5063 (3)	0.3751 (2)	0.0350 (9)	
C20	0.3007 (3)	0.7648 (3)	0.3986 (2)	0.0361 (9)	
H20	0.3568	0.7992	0.4063	0.043*	
C21	0.1510 (2)	0.9574 (3)	0.4023 (2)	0.0352 (8)	
C22	0.1583 (2)	1.0639 (2)	0.3948 (2)	0.0305 (8)	
C23	0.1992 (3)	1.1083 (3)	0.3340 (2)	0.0381 (9)	
H23	0.2259	1.0727	0.2955	0.046*	
C24	0.1997 (3)	1.2072 (2)	0.3315 (3)	0.0392 (9)	
H24	0.2265	1.2367	0.2896	0.047*	
C25	0.1267 (3)	1.2190 (3)	0.4458 (3)	0.0386 (9)	
H25	0.1036	1.2567	0.4854	0.046*	
C26	0.1205 (2)	1.1212 (3)	0.4521 (2)	0.0372 (9)	
H26	0.0919	1.0936	0.4935	0.045*	
O5	0.89687 (19)	0.9980 (2)	0.33618 (19)	0.0566 (8)	
H1W	0.9471	0.9752	0.3683	0.085*	
H2W	0.9002	1.0018	0.2829	0.085*	
O6	0.10303 (19)	0.4671 (2)	0.1547 (2)	0.0618 (8)	
H3W	0.0890	0.4581	0.2031	0.093*	
H4W	0.0571	0.4882	0.1161	0.093*	

### Atomic displacement parameters (Å^2^)

**Table:**

	*U*^11^	*U*^22^	*U*^33^	*U*^12^	*U*^13^	*U*^23^
Hg1	0.03055 (8)	0.02692 (10)	0.04319 (9)	0.00303 (6)	0.01399 (6)	0.00319 (6)
Hg2	0.03092 (8)	0.02783 (10)	0.04418 (9)	0.00243 (6)	0.01377 (6)	0.00243 (6)
Cl1	0.0326 (5)	0.0563 (7)	0.0615 (7)	0.0070 (4)	0.0225 (5)	0.0088 (5)
Cl2	0.0337 (5)	0.0583 (7)	0.0585 (7)	0.0064 (4)	0.0213 (5)	0.0088 (5)
O1	0.0416 (15)	0.0241 (15)	0.0673 (19)	−0.0017 (11)	0.0136 (14)	0.0059 (13)
O2	0.0427 (16)	0.0345 (16)	0.092 (2)	−0.0030 (12)	0.0304 (16)	0.0051 (15)
O3	0.0496 (17)	0.0181 (14)	0.074 (2)	0.0002 (12)	0.0163 (14)	0.0048 (14)
O4	0.0429 (16)	0.0295 (16)	0.081 (2)	−0.0021 (12)	0.0254 (15)	0.0056 (14)
N1	0.0407 (18)	0.0175 (16)	0.0423 (18)	0.0007 (13)	0.0085 (15)	0.0004 (13)
N2	0.0329 (16)	0.0204 (17)	0.0505 (19)	−0.0019 (12)	0.0151 (14)	−0.0009 (13)
N3	0.045 (2)	0.026 (2)	0.047 (2)	0.0007 (13)	0.0098 (16)	0.0011 (14)
N4	0.0443 (19)	0.0212 (17)	0.0430 (19)	−0.0063 (14)	0.0108 (15)	−0.0037 (14)
N5	0.0349 (17)	0.0217 (17)	0.0505 (19)	−0.0045 (13)	0.0135 (14)	−0.0018 (14)
N6	0.0408 (18)	0.0281 (19)	0.050 (2)	0.0005 (14)	0.0120 (16)	0.0004 (15)
C1	0.0333 (19)	0.0218 (19)	0.0300 (19)	0.0068 (14)	0.0103 (15)	0.0038 (14)
C2	0.040 (2)	0.028 (2)	0.037 (2)	0.0015 (16)	0.0096 (17)	0.0025 (16)
C3	0.048 (2)	0.025 (2)	0.057 (3)	0.0112 (18)	0.014 (2)	0.0000 (18)
C4	0.042 (2)	0.041 (3)	0.063 (3)	0.0197 (19)	0.010 (2)	0.004 (2)
C5	0.032 (2)	0.035 (2)	0.054 (3)	−0.0009 (16)	0.0046 (18)	0.0005 (18)
C6	0.036 (2)	0.025 (2)	0.034 (2)	0.0047 (15)	0.0097 (16)	−0.0003 (15)
C7	0.037 (2)	0.025 (2)	0.038 (2)	−0.0028 (15)	0.0117 (17)	−0.0013 (16)
C8	0.036 (2)	0.024 (2)	0.044 (2)	−0.0021 (16)	0.0075 (17)	0.0039 (16)
C9	0.0301 (18)	0.021 (2)	0.037 (2)	0.0014 (14)	0.0052 (15)	0.0032 (15)
C10	0.037 (2)	0.029 (2)	0.044 (2)	−0.0006 (16)	0.0154 (18)	0.0009 (17)
C11	0.039 (2)	0.030 (2)	0.046 (2)	0.0082 (16)	0.0132 (18)	−0.0025 (18)
C12	0.049 (2)	0.037 (3)	0.042 (2)	−0.0021 (18)	0.0186 (19)	0.0061 (19)
C13	0.052 (2)	0.024 (2)	0.044 (2)	0.0024 (17)	0.0164 (19)	−0.0021 (17)
C14	0.0347 (19)	0.023 (2)	0.0320 (19)	0.0054 (14)	0.0118 (16)	0.0031 (15)
C15	0.038 (2)	0.026 (2)	0.033 (2)	0.0030 (15)	0.0092 (17)	0.0014 (15)
C16	0.034 (2)	0.040 (3)	0.057 (3)	0.0023 (17)	0.0062 (19)	−0.0017 (19)
C17	0.038 (2)	0.041 (3)	0.070 (3)	0.0165 (18)	0.006 (2)	0.005 (2)
C18	0.048 (2)	0.026 (2)	0.058 (3)	0.0120 (18)	0.009 (2)	0.0042 (18)
C19	0.041 (2)	0.023 (2)	0.040 (2)	−0.0009 (16)	0.0077 (17)	0.0044 (16)
C20	0.034 (2)	0.028 (2)	0.046 (2)	−0.0025 (16)	0.0108 (17)	−0.0024 (17)
C21	0.031 (2)	0.032 (2)	0.041 (2)	−0.0013 (16)	0.0066 (17)	−0.0004 (17)
C22	0.0282 (18)	0.023 (2)	0.039 (2)	0.0017 (14)	0.0065 (16)	0.0007 (16)
C23	0.047 (2)	0.034 (2)	0.037 (2)	0.0030 (17)	0.0175 (18)	−0.0026 (17)
C24	0.050 (2)	0.026 (2)	0.046 (2)	−0.0040 (16)	0.0194 (19)	0.0040 (17)
C25	0.040 (2)	0.030 (2)	0.048 (2)	0.0069 (16)	0.0135 (19)	−0.0033 (17)
C26	0.034 (2)	0.040 (2)	0.041 (2)	0.0041 (16)	0.0151 (17)	0.0042 (17)
O5	0.0477 (17)	0.068 (2)	0.0577 (19)	0.0121 (15)	0.0196 (15)	0.0016 (15)
O6	0.0449 (17)	0.072 (2)	0.071 (2)	0.0108 (15)	0.0201 (16)	−0.0060 (17)

### Geometric parameters (Å, °)

**Table:**

Hg1—C1	2.059 (3)	C8—C9	1.501 (5)
Hg1—Cl1	2.3189 (9)	C9—C13	1.390 (5)
Hg2—C14	2.058 (3)	C9—C10	1.389 (5)
Hg2—Cl2	2.3231 (10)	C10—C11	1.379 (5)
O1—C2	1.366 (4)	C10—H10	0.9300
O1—H1	0.8200	C11—H11	0.9300
O2—C8	1.233 (4)	C12—C13	1.379 (5)
O3—C19	1.369 (4)	C12—H12	0.9300
O3—H3	0.8200	C13—H13	0.9300
O4—C21	1.237 (4)	C14—C19	1.391 (5)
N1—C7	1.268 (4)	C14—C15	1.402 (5)
N1—N2	1.382 (4)	C15—C16	1.400 (5)
N2—C8	1.354 (4)	C15—C20	1.468 (5)
N2—H2D	0.8600	C16—C17	1.383 (5)
N3—C12	1.340 (5)	C16—H16	0.9300
N3—C11	1.341 (5)	C17—C18	1.379 (5)
N4—C20	1.277 (4)	C17—H17	0.9300
N4—N5	1.386 (4)	C18—C19	1.389 (5)
N5—C21	1.353 (4)	C18—H18	0.9300
N5—H5D	0.8600	C20—H20	0.9300
N6—C24	1.323 (4)	C21—C22	1.502 (5)
N6—C25	1.345 (4)	C22—C23	1.379 (5)
C1—C2	1.388 (5)	C22—C26	1.402 (5)
C1—C6	1.405 (5)	C23—C24	1.387 (5)
C2—C3	1.397 (5)	C23—H23	0.9300
C3—C4	1.384 (5)	C24—H24	0.9300
C3—H3A	0.9300	C25—C26	1.378 (5)
C4—C5	1.389 (5)	C25—H25	0.9300
C4—H4	0.9300	C26—H26	0.9300
C5—C6	1.392 (5)	O5—H1W	0.8363
C5—H5	0.9300	O5—H2W	0.8344
C6—C7	1.473 (5)	O6—H3W	0.8298
C7—H7	0.9300	O6—H4W	0.8293
			
C1—Hg1—Cl1	173.85 (10)	N3—C12—C13	123.8 (3)
C14—Hg2—Cl2	174.67 (10)	N3—C12—H12	118.1
C2—O1—H1	109.5	C13—C12—H12	118.1
C19—O3—H3	109.5	C12—C13—C9	118.5 (3)
C7—N1—N2	116.7 (3)	C12—C13—H13	120.7
C8—N2—N1	117.6 (3)	C9—C13—H13	120.7
C8—N2—H2D	121.2	C19—C14—C15	118.8 (3)
N1—N2—H2D	121.2	C19—C14—Hg2	119.7 (3)
C12—N3—C11	117.0 (3)	C15—C14—Hg2	121.4 (2)
C20—N4—N5	116.5 (3)	C16—C15—C14	119.8 (3)
C21—N5—N4	118.5 (3)	C16—C15—C20	118.0 (3)
C21—N5—H5D	120.7	C14—C15—C20	122.2 (3)
N4—N5—H5D	120.7	C17—C16—C15	120.2 (4)
C24—N6—C25	117.4 (3)	C17—C16—H16	119.9
C2—C1—C6	119.5 (3)	C15—C16—H16	119.9
C2—C1—Hg1	119.4 (3)	C18—C17—C16	120.2 (4)
C6—C1—Hg1	121.1 (2)	C18—C17—H17	119.9
O1—C2—C1	117.6 (3)	C16—C17—H17	119.9
O1—C2—C3	122.0 (3)	C17—C18—C19	119.9 (4)
C1—C2—C3	120.3 (3)	C17—C18—H18	120.0
C4—C3—C2	120.0 (4)	C19—C18—H18	120.0
C4—C3—H3A	120.0	O3—C19—C18	121.7 (3)
C2—C3—H3A	120.0	O3—C19—C14	117.2 (3)
C3—C4—C5	120.2 (3)	C18—C19—C14	121.0 (3)
C3—C4—H4	119.9	N4—C20—C15	120.3 (3)
C5—C4—H4	119.9	N4—C20—H20	119.9
C4—C5—C6	120.2 (3)	C15—C20—H20	119.9
C4—C5—H5	119.9	O4—C21—N5	123.6 (3)
C6—C5—H5	119.9	O4—C21—C22	121.2 (3)
C5—C6—C1	119.8 (3)	N5—C21—C22	115.2 (3)
C5—C6—C7	118.3 (3)	C23—C22—C26	118.4 (3)
C1—C6—C7	121.9 (3)	C23—C22—C21	123.4 (3)
N1—C7—C6	120.6 (3)	C26—C22—C21	118.3 (3)
N1—C7—H7	119.7	C22—C23—C24	118.3 (3)
C6—C7—H7	119.7	C22—C23—H23	120.8
O2—C8—N2	123.6 (3)	C24—C23—H23	120.8
O2—C8—C9	121.0 (3)	N6—C24—C23	124.1 (3)
N2—C8—C9	115.4 (3)	N6—C24—H24	117.9
C13—C9—C10	118.2 (3)	C23—C24—H24	117.9
C13—C9—C8	123.1 (3)	N6—C25—C26	122.9 (3)
C10—C9—C8	118.7 (3)	N6—C25—H25	118.6
C11—C10—C9	119.1 (3)	C26—C25—H25	118.6
C11—C10—H10	120.5	C25—C26—C22	118.8 (3)
C9—C10—H10	120.5	C25—C26—H26	120.6
N3—C11—C10	123.3 (3)	C22—C26—H26	120.6
N3—C11—H11	118.4	H1W—O5—H2W	110.1
C10—C11—H11	118.4	H3W—O6—H4W	111.0
			
C7—N1—N2—C8	163.9 (3)	C8—C9—C13—C12	−179.9 (3)
C20—N4—N5—C21	−166.2 (3)	Cl2—Hg2—C14—C19	2.9 (12)
Cl1—Hg1—C1—C2	−23.9 (11)	Cl2—Hg2—C14—C15	−176.6 (8)
Cl1—Hg1—C1—C6	155.6 (7)	C19—C14—C15—C16	1.2 (5)
C6—C1—C2—O1	177.6 (3)	Hg2—C14—C15—C16	−179.3 (3)
Hg1—C1—C2—O1	−2.8 (4)	C19—C14—C15—C20	178.7 (3)
C6—C1—C2—C3	1.2 (5)	Hg2—C14—C15—C20	−1.8 (5)
Hg1—C1—C2—C3	−179.2 (3)	C14—C15—C16—C17	−0.4 (6)
O1—C2—C3—C4	−177.4 (4)	C20—C15—C16—C17	−178.0 (4)
C1—C2—C3—C4	−1.1 (6)	C15—C16—C17—C18	0.1 (6)
C2—C3—C4—C5	0.8 (6)	C16—C17—C18—C19	−0.6 (6)
C3—C4—C5—C6	−0.6 (6)	C17—C18—C19—O3	178.0 (4)
C4—C5—C6—C1	0.8 (6)	C17—C18—C19—C14	1.4 (6)
C4—C5—C6—C7	179.7 (3)	C15—C14—C19—O3	−178.5 (3)
C2—C1—C6—C5	−1.1 (5)	Hg2—C14—C19—O3	2.1 (4)
Hg1—C1—C6—C5	179.4 (3)	C15—C14—C19—C18	−1.7 (5)
C2—C1—C6—C7	−179.9 (3)	Hg2—C14—C19—C18	178.8 (3)
Hg1—C1—C6—C7	0.5 (5)	N5—N4—C20—C15	−177.1 (3)
N2—N1—C7—C6	178.0 (3)	C16—C15—C20—N4	−179.3 (4)
C5—C6—C7—N1	176.9 (3)	C14—C15—C20—N4	3.2 (5)
C1—C6—C7—N1	−4.2 (5)	N4—N5—C21—O4	4.8 (5)
N1—N2—C8—O2	−4.8 (5)	N4—N5—C21—C22	−175.9 (3)
N1—N2—C8—C9	177.4 (3)	O4—C21—C22—C23	−143.1 (4)
O2—C8—C9—C13	145.8 (4)	N5—C21—C22—C23	37.6 (5)
N2—C8—C9—C13	−36.3 (5)	O4—C21—C22—C26	36.2 (5)
O2—C8—C9—C10	−33.8 (5)	N5—C21—C22—C26	−143.1 (3)
N2—C8—C9—C10	144.1 (3)	C26—C22—C23—C24	−1.0 (5)
C13—C9—C10—C11	1.5 (5)	C21—C22—C23—C24	178.3 (3)
C8—C9—C10—C11	−178.9 (3)	C25—N6—C24—C23	0.5 (6)
C12—N3—C11—C10	2.0 (5)	C22—C23—C24—N6	1.1 (6)
C9—C10—C11—N3	−2.5 (5)	C24—N6—C25—C26	−2.2 (5)
C11—N3—C12—C13	−0.6 (5)	N6—C25—C26—C22	2.3 (5)
N3—C12—C13—C9	−0.2 (6)	C23—C22—C26—C25	−0.6 (5)
C10—C9—C13—C12	−0.3 (5)	C21—C22—C26—C25	−179.9 (3)

### Hydrogen-bond geometry (Å, °)

**Table:**

*D*—H···*A*	*D*—H	H···*A*	*D*···*A*	*D*—H···*A*
O6—H4W···O2^i^	0.83	2.15	2.898 (4)	150
O6—H3W···O3	0.83	2.30	3.023 (4)	146
O5—H2W···O1	0.83	2.17	2.963 (4)	159
O5—H1W···O4^ii^	0.84	2.06	2.876 (4)	166
N5—H5D···O6^iii^	0.86	2.04	2.872 (4)	162
N2—H2D···O5^iv^	0.86	2.06	2.890 (4)	161
O3—H3···N6^v^	0.82	1.92	2.737 (4)	171
O1—H1···N3^vi^	0.82	1.93	2.733 (4)	167

Symmetry codes: (i) *x*−1, *y*, *z*; (ii) *x*+1, *y*, *z*; (iii) −*x*+1/2, *y*+1/2, −*z*+1/2; (iv) −*x*+3/2, *y*−1/2, −*z*+1/2; (v) *x*, *y*−1, *z*; (vi) *x*, *y*+1, *z*.
